# Are universities left‐wing bastions? The political orientation of professors, professionals, and managers in Europe

**DOI:** 10.1111/1468-4446.12716

**Published:** 2019-12-10

**Authors:** Herman G. van de Werfhorst

**Affiliations:** ^1^ Department of Sociology University of Amsterdam Amsterdam the Netherlands

**Keywords:** cultural values, economic values, Europe, political orientation, professors, social class

## Abstract

Universities are accused of being left‐wing bastions, unwelcoming to conservative and right‐wing professors. However, we know little about the political orientation of professors in comparison to other professionals, which would be the right comparison group if we want to know whether universities are potentially hostile environments to conservatives. Examining culturally and economically oriented political orientations in Europe, it is demonstrated that professors are more liberal and left‐leaning than other professionals. However, there is no greater homogeneity of political orientations among the professoriate relative to other specific professions, suggesting that there is a diversity of opinions which is similar to what professionals would find in other occupations. One exception concerns attitudes towards immigration, on which professors have more liberal orientations and comparatively low residual variance around that more liberal mean. Importantly, the difference between professors and other professionals is not so clear within graduates from the social sciences, but emerges more clearly among graduates with a medical, STEM, economics or law degree. An important political cleavage exists between professionals and managers, a group of similar social standing.

## 1 INTRODUCTION

In various societies debates have arisen on the lack of political diversity among academics in universities.^1^ Critics have argued that one‐sided political views in the university, particularly leaning to left‐wing or liberal orientations, may prevent the teaching of a diverse set of opinions and worldviews, and may constrain academics who have unconventional views to express themselves and follow their own research interests. Recent scholarship suggests that academic social psychologists became increasingly liberal (Duarte et al., 2015), and conservatives find it less attractive to opt for an academic career (Gross & Fosse, 2012). But criticism does not only come from the political right: academic freedom has also been criticized by critical theorists as serving the interest of social elites (Williams, 2016). A non‐diverse university may be threatening to academic freedom when researchers do not feel free to investigate following their own interest, while academic freedom is essential for scientific progress (Williams, 2016).

While evidence exists that academics, on average, have more left‐leaning orientations than the general population (Gross & Fosse, 2012; Klein, Stern, & Western, 2005; Ladd & Lipset, 1975), the presumed *homogeneity* in political orientations among professors has not yet been properly investigated. Moreover, academics have not been compared to the most evident comparison group of (other) professionals, which is unfortunate because the social class of professionals is known to be more egalitarian and liberal than the social class of managers—a group of similar occupational standing in the standard sociological social class literature (Brint, 1984; Güveli, Need, & De Graaf, 2007; Kalmijn & Kraaykamp, 2007; Van de Werfhorst & De Graaf, 2004).

It is important to compare professors to other professionals, both in their average political orientation and within‐group diversity in orientations, because universities are thought to be organizations hostile to conservative or right‐wing scholars. If universities are left‐wing bastions where people with divergent (non‐liberal) orientations would feel unwelcome, we would not only expect that professors are more left‐leaning than other professionals (with similar levels and fields of education), but also that there is a comparatively small dispersion around that more left‐wing average orientation, leaving little room for diversity. If the selection process into the professoriate were biased against scholars with divergent opinions, a homogenizing process would have taken place beyond what one may expect from the self‐selection into specific fields of study and the potential causal effect of field of study on political orientations. I consider this homogeneity‐inducing process a central claim of the thought that universities are left‐wing bastions intolerant to a diverse set of opinions. Previous scholarship has not been concerned with within‐group homogeneity among professors and comparison groups, although low variability of values within a profession have been defended as resulting from scientific wisdom rather than bias (Fuller & Geide‐Stevenson, 2014).

This paper studies the left‐wing bastion hypothesis by examining political orientations of people in various occupational groups within the classes of professionals and managers, both in terms of the group averages and within‐group homogeneity. Inspired by contemporary class theories and Bourdieusian field theory, I argue that occupational groups and educational background structure political orientations, causally and through processes of selection. This is likely not only the case for professors, but also for other occupational groups. Occupations are examined that are typically seen as rich in cultural capital (professors, artists), rich in economic capital (e.g., CEOs of large private enterprises), and occupations in which there is no clear dominance of one type of capital over the other (engineers). 

Using large‐scale representative survey data of the European Social Survey I compare the political orientation of professors with that of other professionals, managers, and other groups of workers in Europe. Using four indicators of political orientation, I cover both the economic and cultural dimensions of political cleavages (Van der Brug & Van Spanje, 2009). While the general question on self‐placement is informative about general political tendencies, the underlying cultural and economic dimensions of political orientation can reveal more specifically on which issues professors potentially deviate from other occupational groups.

### 1.1 Occupations and political orientation

Back in the 1970s it was already established that American professors were more strongly connected to the Democratic Party than to the Republican Party (Ladd & Lipset, 1975). Moreover, the proportion of supporters of the Democrats is larger than in the American population as a whole, suggesting that there is a mis‐representation of political ideologies in college campuses (Gross, 2013). However, the criticism to universities as left‐wing bastions does not only concern the distribution of ideologies, but also the closure of the occupational group towards outsiders having different viewpoints. This can be assumed to have led to a homogenization process among the professoriate, leading to low dispersions around a left‐wing/liberal orientation. Sociological theory may help us to examine whether and why professors are an exceptional occupational group with regard to political and cultural orientations.

New class theory highlights an important change in the class structure with the rise of post‐industrial society, with a new class of knowledge workers that differ from the traditional “ruling” class of proprietors (Brint, 1984). While the influential Erikson‐Goldthorpe class schema sees the service class as one class that includes managers and professionals, alternative class schemas have separated them—for example, “experts” in the work of Wright (1997) or the modern and traditional professionals in the approach by Savage et al. (2013). More refined approaches distinguished knowledge workers or socio‐cultural specialists as a particular group of professionals that would have strongly different political views (Brint, 1984; Güveli et al., 2007; Van de Werfhorst & De Graaf, 2004). From this literature it becomes evident that, in order to judge whether professors are exceptionally left‐leaning, one would need to compare them to other professionals rather than to the population as a whole. If professors have political orientations similar to other professional groups, it is not likely that universities as organizations are hostile to people with divergent attitudes.

Further disaggregations of social class are proposed by recent developments in class theory that posit that many of the processes that manifest classes take place at the occupational (“micro‐class”) level rather than at the “big class” level (Grusky & Sørensen, 1998; Weeden & Grusky, 2005). Inspired by the relevance that Durkheim attached to occupational organizations as intermediaries between the individual and society, occupational class theory proposes a number of homogenization mechanisms happening at the level of occupations: the *allocation* of people into positions, the *social conditioning* of workers (e.g., through training, closure, and interest formation), and the *institutionalization of conditions* such as structuring and rewarding work (Weeden & Grusky, 2005). Given the relevance of occupational groups as evidenced in this approach (not just of professors), it is likely that such homogenizing processes happen in other occupational groups as well, certainly when members of an occupational group are well integrated into formal (professional) associations, like judges, architects, and accountants. The disaggregated approach has proven relevant for many outcomes, including lifestyles, political orientations, and social mobility (Weeden & Grusky, 2005; Van de Werfhorst & Luijkx, 2010).

The disaggregated approach to class resonates with social field theory. Professions can be seen as working in a field that structures the orientations towards the world. Regularities in individual action (such as the coherent preference for a particular political ideology within a professional group) can, according to field theory, be understood “in recourse to position vis‐à‐vis others” (Martin, 2003, p. 1). An important classification scheme in field theory is in the types of capital that are associated to the social location of occupations, in particular economic and cultural capital (Bourdieu, 1984). Depending on the dominance of a particular type of capital, some occupational groups are richer in cultural capital, while others are richer in economic capital (Bourdieu, 1984; De Graaf & Kalmijn, 2001). Academics—together with, for example, artists and journalists—are typically seen as part of the cultural elite. CEOs of large organizations are, by contrast, part of the economic elite in which the dominant form of capital is of the economic type.

The structuring character of elite formation is already exemplified in the selection of the *grandes écoles*; the elite institutions in the French system of higher education (Bourdieu, 1998). Social classes differ in their preference for the separate schools that exist for, for instance, public administrators, business elites, and social and humanistic sciences. Enrolment into a particular institution does not only structure labour market opportunities, but also the political and cultural orientations of graduates. Elite institutions are thus part and parcel of the professional field in which graduates integrate (Van Zanten, 2009).

While field theory would thus assume that each profession has its own processes of identification and orientation, in which the position of one’s own profession is delineated in relation to others, there is also a special position of professors according to Bourdieu (1988). Professors share with artists a dominance of cultural over economic capital, and can in this respect be positioned against managers of large corporations. However, they have a more stable employment relationship, with regular incomes and more traditional life orientations than artists. However, there are clear differences between faculties. Professors in the fields strongly connected to scientific honor (reflected in national academic board memberships and indicators of academic prestige) are strongly connected to cultural capital (the arts, humanities, but also physics and mathematics), and have corresponding, liberal orientations. Professors in more powerful fields of medicine and law have more traditional orientations.

The question is, however, whether these differences reflect variations in the fields of study people have been educated in, or whether there is a true “battle” between faculties as organizations with their own interests, as Bourdieu (1988) would argue. Differences across graduates from different fields of study (independent of their profession) have often been demonstrated. Fields of study are not only correlated to career progression (Jacob & Klein, 2019), but also to wider political and cultural values (Van de Werfhorst & Kraaykamp, 2001). While professors in the social sciences and humanities are often found to be particularly left‐wing compared to professors in other fields (Gross, 2013; Klein et al., 2005), it is unclear whether this is true when these professors are compared with other professionals who took a social science or humanities degree. If the left‐wing bastion hypothesis would be true, faculties of social and humanistic sciences would be particularly hostile to divergent (non‐liberal) opinions of potential faculty members. Under such a regime, it is likely that conservatives would find it less and less attractive to opt for an academic career (Gross & Fosse, 2012). If, on the other hand, political orientations are correlated to the field of study people enrolled in, independent of whether they became professors or found jobs outside the university, then this could be explained by selection effects into these fields or causal effects of the fields on political orientations. There would, in that case, be little to worry about the presumed social closure of the professoriate whose interest it would be to exclude conservative or right‐wing scholars.

Inspired by these literatures, the current paper studies political orientations of a range of occupational groups that differ in the amount of economic and cultural capital. Professors are compared to other professionals and managers, as the main categories of elites that vary in the type of capital (economic or cultural) that is dominant for their location in the social space. This is done for graduates within the same field of study. A crucial test of the left‐wing bastion hypothesis as I see it, is whether professors do not only differ with regard to the *average* political orientation, but also in its *dispersion*. Variations within occupational and educational groups have been highlighted before. One example is the variability in values among economists inside and outside academia (Block & Walker, 1988; Van Dalen, 2019). Achievement‐oriented economists with little involvement with the public interest are more supportive of income inequality and more critical to immigration than publicly concerned economists who score low on achievement‐oriented values, although other values, such as opposition against tariffs, were widely shared among economists. Also, students obtaining a teaching qualification varied in their political cynicism depending on their cohort, and varied in what they considered good citizenship (Wilkins, 1999). Furthermore, the political orientation of CEOs is associated to the existing pay differences within the management team in their organizations (Chin & Semadeni, 2017).

**Hypothesis**: According to the left‐wing bastion hypothesis, we would expect a more homogeneous, and more left‐leaning orientation among professors relative to other professional occupations (such as lawyers, architects, computer programmers, accountants, engineers, and medical doctors), who have similar educational histories but different career paths after leaving education. Following Bourdieu’s metaphor of a battle between faculties, one would furthermore expect that professors in the humanities and social sciences stand out as particularly (homogeneously) left‐wing, compared to other professors, also in relation to the non‐professors with similar educational backgrounds. Also, important differences are to be expected between professionals and managers or large corporations, as groups that vary in the dominance of cultural and economic capital.

If, however, professors differ little from other professionals or CEOs with similar educational histories, the professoriate would not stand out as a closed bastion intolerant to divergent opinions. Such structuration patterns by occupational groups are interesting from the perspective of social class theory as it has been argued that class formation and identification increasingly take place at the occupational level rather than at the level of broad social classes (Weeden & Grusky, 2005). But such cleavages would be less worrying in relation to the concern that universities are closed organizations hostile to employees with atypical political orientations.

## 2 RESEARCH DESIGN

### 2.1 Data

Similar to the study by Gross and Fosse (2012), I use general population surveys to identify professors and comparison groups. I make use of the European Social Surveys (ESS). The ESS data have been collected biannually since 2002 (as a repeated cross‐sectional study), and is part of the core European Research Infrastructure funded by the European Commission. We use rounds 1–8 for this analysis (the Cumulative File rounds 1–7 edition 1 to which the round 8 Integrated File edition 2.0 has been added), and selected respondents aged 25–65 with a valid occupation code (ESS, 2016, 2018). This gives us an analytical sample of *N* = 234,306 (with slightly fewer observations in the regression models depending on the valid *N* on the dependent variables).

There are several advantages of using the ESS. First, thanks to rigorous sampling procedures and efforts, the response rates are comparatively high (the ESS works with a target response rate of 70%; most countries have response rates between 50 and 70%). Second, the ESS takes great effort in collecting high‐quality socio‐demographic information including educational attainment and respondent’s occupation. Third, the ESS is primarily aimed at measuring social and political attitudes, which enables us to study an important set of political attitudes of occupational groups. Fourth, the ESS Core Team has developed extensive weighting procedures of which we make use (ESS, 2014). We weight the data using the design weight *DWEIGHT* (correcting for differential probabilities of individuals of being sampled due to each country’s specific sampling design) and the population size weight *PWEIGHT* (so we can generalize to the overall European population across the countries in our database). For within‐country descriptions (Table 2) we only used the *DWEIGHT* variable. The data we use includes 31 countries (see Table 2 for a list of countries). There was one country without one single professor in the sample (Turkey), and we omitted that country from the data file for that reason. As the educational field of study of respondents is only known in ESS rounds 2–4, we restricted the analyses by field only to these rounds (*N* = 80,179).

### 2.2 Background variables

Occupational group is identified on the basis of the International Standard Classification of Occupations (ISCO) version 1988 (ESS waves 1–5) and version 2008 (ESS waves 6–8), referring to the respondent’s current occupation, or the last occupation if the respondent is currently not employed. The first digit of this four‐digit classification distinguishes managers (first digit 1), professionals (2), and “other occupations” (3–9). Within the class of professionals, university professors are identified by code 2310 (“College, university and higher education teaching professionals”). Excluded therefore are university researchers without a teaching responsibility and non‐academic personnel, and included is everybody whose main job involves teaching in higher education (including tenured professors of all ranks, and lecturers with temporary and/or part‐time contracts such as adjunct professors and teaching assistants). As our interest is in the comparison of professors to other professionals and managers, the group of “other occupations” (i.e., non‐professionals and non‐managers) is not further disaggregated but serves as overall comparison to our groups of interest. Additional analyses make a distinction in various professional occupational groups. Table 1 gives the detailed occupational codes to identify all occupational groups that were investigated. As said, the identified occupations represent different locations in the social space of occupations as presented in Bourdieu’s work (Bourdieu, 1984). Besides professors, teachers and artists are also identified as high‐cultural capital occupations. Other professional groups are stronger on economic capital, such as accountants, while still others score reasonably high on both kinds of capital without any clear dominance of either (medical doctors, engineers). Within the group of managers, relevant distinctions are between legislators, which would, in Bourdieu’s depiction of senior civil servants, possess a dominance of cultural capital, and the CEOs of large organizations, which are typically seen as a group with a dominance of economic capital. It should be noted that the list of occupations is not exhaustive, but identifies clearly positioned occupational groups sufficiently large to use for the empirical analysis.

**Table 1:** Occupational codes to identify occupational groups

	ISCO 1988	ISCO 2008	Weighted frequency
Managers	1	1	18,773
Of which			
Legislators and senior officials of special interest groups	1000–1133	1100–1114	449
Managers of large organizations, CEOs	1200–1239	1000, 1120–1349	11,493
Managers in small and medium enterprises (SMEs)	1300–1319	14	6,831
Professionals	2	2	41,077
Of which			
Professors	2310	2310	1,306
Teachers and teacher associates	232–235, 33	232–235, 5312	13,894
Engineers	2143, 2144	215	4,176
Medical doctors	2221	221	1,579
Computer programmers	213	25	2,338
Accountants	2411	2411	1,865
Architects, town and traffic planners	2141	2161, 2162, 2164	1,060
Lawyers and judges	242	261	1,211
Artists	245	264, 265	1,577
Other professionals			12,071
Armed forces	100	0, 100, 110, 210, 300, 310	882
Other occupations	3–9	3–9	173,574

Note

The ISCO codes identify occupations at the four digit level. The number of digits in the table determines at which level of detail the occupational groups were identified. Higher‐order digits can be filled. For example: 1 indicates all occupational codes starting with a 1, 213 indicates all occupations starting with 213. The provided codes are the ones seen in the ESS data.

**Table 2:** The proportion of professors in each country and ESS round

Country	ESS round							
	1 (2002)^a^	2 (2004)	3 (2006)	4 (2008)	5 (2010)	6 (2012)	7 (2014)	8 (2016)
Austria	0.003	0.002	0.002				0.009	0.002
Belgium	0.002	0.000	0.010	0.008	0.004	0.008	0.006	0.003
Britain	0.011	0.005	0.013	0.013	0.008	0.007	0.003	0.006
Bulgaria			0.002	0.001	0.002	0.002		
Croatia				0.005	0.005			
Cyprus			0.000	0.004	0.003	0.000		
Czech Republic	0.001	0.001		0.002	0.001	0.003	0.003	0.002
Denmark	0.005	0.006	0.010	0.005	0.009	0.016	0.010	
Estonia		0.002	0.005	0.003	0.005	0.005	0.006	0.003
Finland	0.006	0.004	0.006	0.003	0.003	0.004	0.009	0.005
France	0.007	0.010	0.004	0.010	0.017	0.002	0.006	0.007
Germany	0.002	0.004	0.001	0.003	0.003	0.004	0.003	0.005
Greece	0.000	0.001		0.001	0.001			
Hungary	0.003	0.010	0.002	0.001	0.003	0.000	0.001	0.000
Iceland		0.000				0.002		0.009
Ireland	0.007	0.007	0.012	0.010	0.007	0.004	0.002	0.005
Israel	0.008			0.016	0.008	0.008	0.010	0.009
Italy	0.005	0.003				0.011		0.004
Lithuania					0.007	0.004	0.004	0.004
Luxembourg	0.006	0.006						
Netherlands	0.007	0.001	0.005	0.006	0.001	0.008	0.009	0.005
Norway	0.010	0.005	0.013	0.005	0.007	0.007	0.008	0.009
Poland	0.007	0.003	0.001	0.007	0.002	0.004	0.001	0.002
Portugal	0.002	0.002	0.002	0.005	0.001	0.007	0.003	0.006
Russia			0.005	0.008	0.003	0.002		0.004
Slovakia		0.005	0.004	0.007	0.001	0.002		
Slovenia	0.005	0.006	0.006	0.004	0.006	0.007	0.001	0.003
Spain	0.006	0.018	0.002	0.007	0.011	0.004	0.003	0.004
Sweden	0.007	0.005	0.004	0.008	0.007	0.009	0.006	0.015
Switzerland	0.003	0.002	0.005	0.002	0.005	0.008	0.003	0.002
Ukraine		0.004	0.005	0.007	0.007	0.005		

Notes

Weighted data (sampling design weight *DWEIGHT*). Empty cells indicate that the country did not take part in the respective ESS round.

a These are the modal survey years for each wave, although some countries have collected data in adjacent years.

Table 2 presents the proportion of professors in each of the countries in each of the ESS rounds. It shows that, after applying weights, the surveys have a proportion of professors (or more precisely: anyone with a teaching function in higher education) between 0 and 1.8%. The overall weighted average across the 31 European countries is 0.53%, so roughly 1 in 188 individuals with a current or past occupation. As a check we examined the population distribution of the Netherlands in 2016, one of the included countries with reliable employment statistics through the *Vereniging van Samenwerkende Nederlandse Universiteiten* (VSNU) that represents the research universities, and the *Vereniging Hogescholen* (VH) that represents universities of applied science (which offer bachelor degrees). In total, 26,864 academics worked in Dutch universities as a PhD candidate, other scientific personnel, or as an assistant/associate/full professor,^2^ and 30,995 employees in the category of “teaching and researching personnel” in the universities of applied science.^3^ In total this is 57,819, which includes individuals counted twice because they work both at a research university and a university of applied science. Set off against the total labor force of 8,941,500,^4^ this gives us a proportion of 0.006 (while it is 0.005, or 0.5%, in the ESS, Table 2). With the possibility of double counts and the inclusion of non‐teaching researchers in the population data, the proportion of 0.5% professors does not seem unrealistic.

The field of study of the highest completed level of education is added to the model. In ESS rounds 2–4, field of study is asked as follows: In which one of these fields or subjects is your highest qualification? The ESS variable *EDUFLD* is more detailed than we examine here; it has 14 categories which were recoded into 6 broad fields. It includes a category “general/ no specific field”, typically identifying primary and non‐vocational secondary education. Table 3 shows how educational field of study was coded. Most fields are fairly unequivocally classified, including the humanities, the STEM (science, technology, engineering and mathematics) fields, the (para‐) medical field, and the legal field. The social sciences are, importantly, separated from the category of economics and business. This represents the faculty ordering in many European countries, while economics would sometimes be seen as a social science in the American context. I classified the field of personal care services in the social sciences. While this field is a bit broad in coverage (and small in numbers under the college graduates), it includes studies like domestic science, and catering.^5^

**Table 3:** Classification of fields of study

Field of study	ESS category	Weighted frequency	%
Humanities/arts	Art, fine/applied	4,595	5.7
	Humanities		
STEM (Science, Technology, Engineering and Mathematics)	Technical and engineering	25,782	32.2
	Agriculture/forestry		
	Science/mathematics/computing etc.		
	Transport and telecommunications		
Social Sciences	Teacher training/education	13,436	16.8
	Social studies/administration/media/culture		
	Personal care services		
(Para‐) Medical	Medical/health services/nursing etc.	5,853	7.3
Economics/Business	Economics/commerce/business administration	10,325	12.9
Legal	Law and legal services	2,213	2.8
	Public order and safety		
	[General/no specific field]	17,975	22.4
Total		80,179	100.0

Note

Field of study is only available in the ESS rounds 2, 3 and 4.

The models control for educational attainment, which is measured with a set of dummy variables (less than lower secondary, lower secondary, upper secondary, some post‐secondary, college degree). We furthermore control for sex (male = 1, female = 0), age and age squared (to adjust for possible non‐linear age correlations with political orientation).^6^ We furthermore added fixed effects for country and ESS round, to filter out baseline differences in political orientations between societies and time periods. Note that we present marginal effects plots summarized for individuals with a college degree, and the mean value on other control variables (i.e., the mean between men and women, with mean age and mean age squared, of the average country and average ESS round). For the graphs by field of study we omitted the category of “general/no specific field”, although that category is included in the model.

### 2.3 Assessing political orientations

While political preferences are expressed most clearly in the voting booth, party choice is an incomplete measure of political bias among professors. There is a lot of variability in orientations among professors with similar party identifications in the U.S.A. (i.e., Democrat or Republican; Klein & Stern, 2008). Moreover, electoral systems differ widely between European societies. A more appropriate way for our purposes to analyze political preferences is to investigate political attitudes. In the political sciences two political axes are usually distinguished to identify political orientation: an economic dimension of left versus right (with the left identifying as pro‐redistribution of incomes and a strong welfare state, and the right characterized by pro‐market attitudes and preferences for low taxes), and a cultural “GAL‐TAN” dimension (Green‐Alternative‐Libertarian vs. Traditional‐Authoritarian‐Nationalist, sometimes called the libertarian‐authoritarian dimension, Bakker et al., 2015). Research has shown that, over time, the right‐left political identification has increasingly been based on cultural (or GAL‐TAN), rather than economic (or traditional left‐right) issues (Baldassarri & Gelman, 2008; Vries, Hakhverdian, & Lancee, 2013). Moreover, this trend is partly due to cohort replacement as the political right‐left orientation of younger cohorts is more strongly driven by cultural issues (Rekker, 2016). Also the link between political ideology and lifestyles is increasing (DellaPosta, Shi, & Macy, 2015).

There is some debate about the question whether political values are consistent across domains. Especially in the 1960s–1980s, political values were thought to be inconsistent across domains (Wuthnow, 2008). As Baldassarri and Gelman (2008) show, the connection between political ideology and values became stronger across time in the United States, while there is no trend towards stronger correlations across issue domains. Also British research showed that political values were in fact highly internally consistent, and can be conceptualized in the two dimensions that are conceptualized here (Evans, Heath, & Lalljee, 1996). The tolerance towards immigration and to further European integration can be seen as part of the GAL‐TAN dimension (Hooghe, Marks, & Wilson, 2002), although both political views can of course also be driven by economic motivations. In any case, economic redistribution, immigration and further European integration are important issues in the contemporary political debates in Europe. I created *z*‐scored variables on the analytical sample, across countries and waves.

A general *right‐left self‐placement* is examined using the survey question “In politics people sometimes talk of ‘left’ and ‘right’. Using this card, where would you place yourself on this scale, where 0 means the left and 10 means the right?” (The variable was recoded such that higher scores indicate a left‐wing orientation.)

Second, the opinion on the role of the government in economic redistribution is asked by the survey question “The government should take measures to reduce differences in income levels”, where respondents could answer on a 1–5 scale with answer categories “Agree strongly”, “Agree”, “Neither agree nor disagree”, “Disagree”, “Strongly disagree” (the variable was recoded such that higher scores indicated a pro‐redistribution attitude).

The third indicator is tolerance to immigration to one’s own country of residence. This is assessed with the following questions: “Would you say it is generally bad or good for [country]’s economy that people come to live here from other countries?” (11‐point scale from 0 “Bad for the economy” to 10 “Good for the economy”); “Would you say that [country]’s cultural life is generally undermined or enriched by people coming to live here from other countries?” (11‐point scale from 0 “Cultural life undermined” to 10 “Cultural life enriched”); and “Is [country] made a worse or a better place to live by people coming to live here from other countries?” (11‐point scale from 0 “Worse place to live” to 10 “Better place to live”). Cronbach’s alpha for this scale was 0.85, indicating a high reliability. The average was taken over the standardized items, in a way that a high score indicates tolerance to immigration.

The last indicator is people’s support for further European integration. This is assessed with a single item: “Now thinking about the European Union, some say European unification should go further. Others say it has already gone too far. Using this card, what number on the scale best describes your position?” (11‐point scale from 0 “Unification has already gone too far” to 10 “Unification go further”). This question was not asked in ESS rounds 1 and 5.

We *z*‐standardized each of the outcome variables across the whole dataset, with mean 0 and standard deviation 1. Table 4 shows the descriptive statistics of the used variables, following listwise deletion with regard to independent variables occupation, education, gender and age (the variance function regression models do not allow us to use multiple imputation).

**Table 4:** Descriptive statistics

	Valid *N*	Mean	Standard deviation	Minimum value	Maximum value
Right‐left self‐placement	199,375	0	1	−2.382	2.134
Government should reduce income differences	231,316	0	1	−2.721	1.070
Tolerant to immigration	231,555	0	1	−2.475	2.477
Further European integration	150,616	0	1	−1.879	1.920
Education	234,306	3.463	1.272	1	5
Gender (male = 1, female = 0)	234,306	0.473	0.499	0	1
Age	234,306	45.248	11.489	25	65
ESS round	234,306	4.508	2.230	1	8

Note

Unweighted data.

### 2.4 Modeling strategy

Given our interest in the group means and the dispersions around the predicted group means, I make use of a joint set of equations predicting (1) the mean score on political outcomes, and (2) the variance in residuals of the mean equation. I follow the variance function regression approach proposed by Western and Bloome (2009). Fixed effects are added for country and ESS round. The residual variance informs us about the dispersion of attitudes around the predicted mean. The left‐wing bastion hypothesis would assume that professors are more left‐wing (higher average), and have lower dispersions (lower residual variance around the group mean) than other professionals.

If the variance of the residuals is a function of predictor variables (e.g., occupational group), there is heteroskedasticity. This violates the homoskedasticity assumption of ordinary least squares regression models, leading to biased estimates of the standard errors. The variance function regression model relaxes the homoskedasticity assumption.

A common solution for heteroskedasticity is reweighting the data by assigning lower weights for individuals with higher residuals. The Western and Bloome approach takes the following steps (following the notation of Western & Bloome, 2009, p. 301), with $y_{i}$ standing for the individual score on the outcome variable (political orientation), $x_{i}$ standing for the predictor variables of the linear regression model, and $z_{i}$ for the predictor variables of the variance function regression:

1. First estimate a linear regression model of $y_{i}$ on $x_{i}$, providing estimated coefficients $\hat{\beta }$ and residuals $\varepsilon_{i}=y_{i}-{x^{\prime }}_{i}\hat{\beta }$.
2. Then fit a gamma regression with log link of $\varepsilon_{i}^{2}$ on $z_{i}$, yielding current estimates $\hat{\lambda }$. Then save the fitted values ${\hat{\sigma }}_{i}^{2}=\exp\left({z^{\prime }}_{i}\hat{\lambda }\right)$.
3. Fit a *weighted* linear regression model of $y_{i}$ on $x_{i}$, with weights determined by the inverse of the individual squared residual $1/{\hat{\sigma }}_{i}^{2}$. After this model, update the saved residual ${\hat{\varepsilon }}_{i}$, and evaluate the log‐likelihood.
4. Iterate steps 2 and 3 to convergence (i.e., no improvement of model fit in terms of log‐likelihood), updating $\hat{\beta }$ and ${\hat{\varepsilon }}_{i}$ from the weighted linear regression, and $\hat{\lambda }$ and ${\hat{\sigma }}_{i}^{2}$ from the gamma regression.^7^

Heteroskedasticity can point to an incomplete specification of the basic linear regression model, for instance through omitted variables or functional form. For our purposes it is not very important whether heteroskedasticity (in our case: different levels of dispersion within occupational groups) results from omitted variables or not. If, for example, there would be larger homogeneity among professors than among other professionals because of a particular distribution of intelligence, there would still be a situation in which people with divergent opinions may find it hard to find their place in the university.

The model predicting the mean outcome has the following form, with *G* + 1 occupational groups, 5 educational levels, 32 countries, and 8 ESS rounds (for these nominal and ordinal variables one category was omitted):

(1) $$y_{i}=\alpha +\beta_{1.1-1.G}\;\text{occupational group}+\beta_{2}\;\text{male}+\beta_{3.1-3.4}\;\text{educ}+\beta_{4}\;\text{age}+\beta_{5}\;{\text{age}}^{2}+\beta_{6.1-6.31}\;\text{country}+\beta_{7.1-7.7}\;\text{ESS round}+\varepsilon_{i}.$$

**Figure 1:**
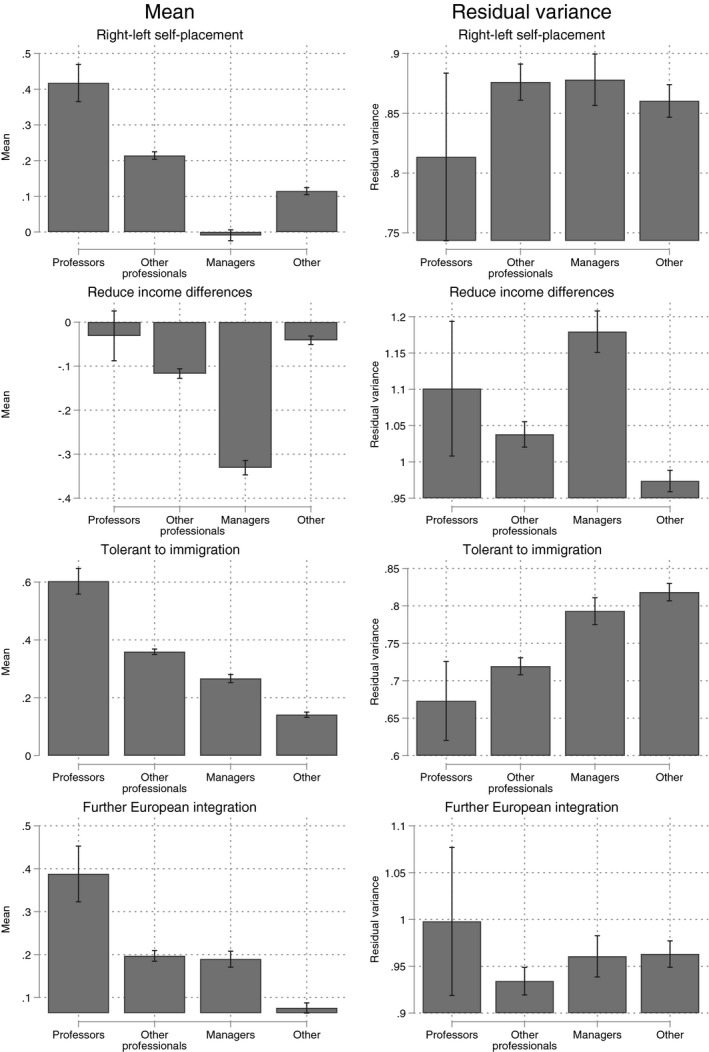
Predicted mean and residual variance around the predicted mean by occupational group, all non‐professoriate professionals in one group. Confidence intervals (95%) are displayed with the black vertical lines. Marginal means are shown for people with a college degree, average age, average ESS round and the average country

The variance function regression model has the following form:

(2) $$\varepsilon_{i}^{2}=\alpha +\lambda_{1.1-1.G}\;\text{occupational group}+\lambda_{2}\;\text{male}+\lambda_{3.1-3.4}\;\text{educ}+\lambda_{4}\;\text{age}+\lambda_{5}\;{\text{age}}^{2}+\lambda_{6.1-6.31}\;\text{country}+\lambda_{7.1-7.7}\;\text{ESS round}+\xi_{i}$$

**Figure 2:**
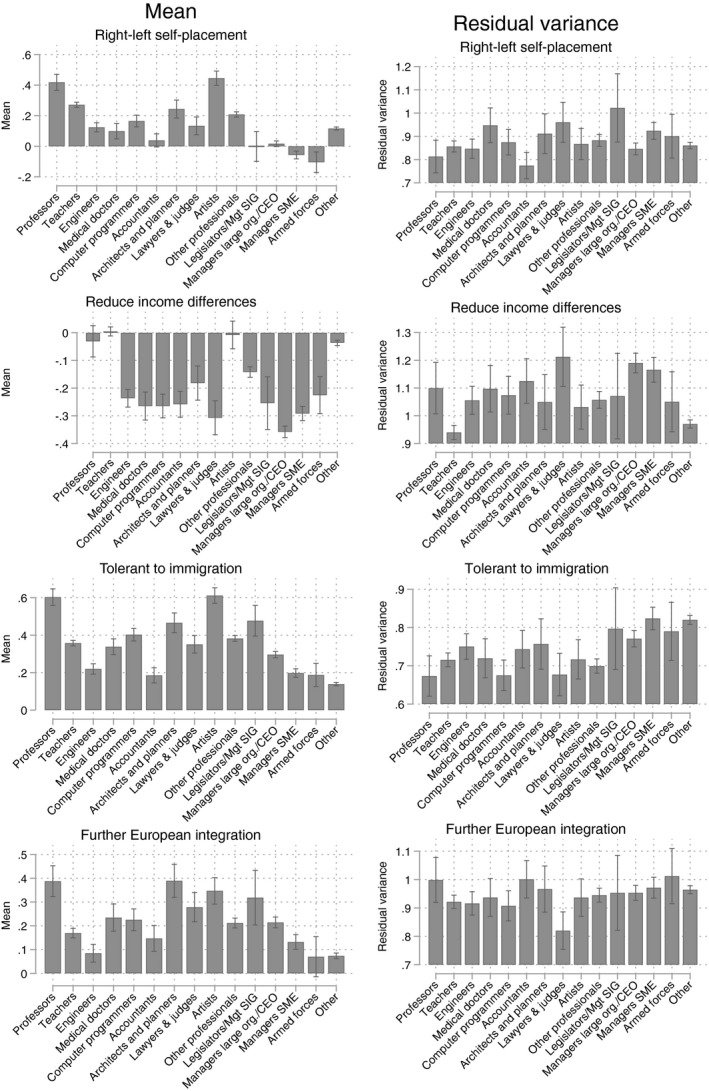
Predicted mean and residual variance around the predicted mean for specific professions and other occupational groups. Confidence intervals (95%) are provided with the black vertical lines. Marginal means are shown for people with a college degree, average age, the average ESS round and the average country

The models by field of study (see Figure 2 later) also add the interaction term between fields of study and occupational group (including main effects).

**Figure 3:**
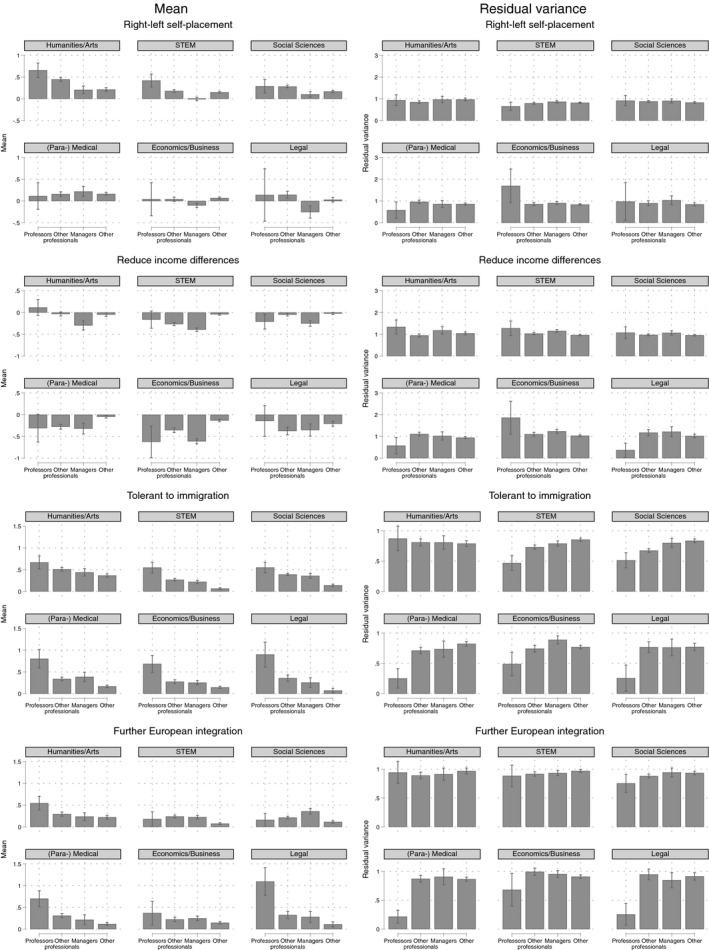
Predicted mean and residual variance around the predicted mean by occupational group and field of study in tertiary education. Confidence intervals (95%) are displayed with the black vertical lines. Marginal means are shown for people with a college degree, average age, the average country, and the average survey year. STEM stands for science, technology, engineering and mathematics

## 3 RESULTS

The tables with the estimates can be found in the Appendix. Marginal plots are used to display the most relevant results. Figure 1 shows that professors are more left‐leaning than other professionals on all four indicators. On right‐left self‐identification, attitudes to immigration, and support for further European integration the difference with other professionals is around 0.2 standard deviations. Professors are also more egalitarian on the question whether governments should reduce income differences, with a difference of around 0.1 standard deviations. It should be noted that professors score around the mean score across the total distribution of economic redistribution attitudes (value 0), given that the effects are identified for people with college education, who are less egalitarian than people with lower levels of education.

With regard to the variance around the predicted occupational group mean, the evidence for the left‐wing bastion hypothesis is mixed; the variance is (somewhat) lower among professors than among other professionals on right‐left self‐identification, and attitudes towards immigration, but the difference is not significant. With regard to income redistribution and further European integration there is no greater homogeneity among professors than among other professionals. Note that the group of other professionals is very diverse, and we examine more specific professional groups below.

An important political cleavage exists between professors and professionals on the one hand, and managers on the other. On all four indicators professors are more liberal than managers, and for professionals this holds for three of the four variables (except further European integration). The gap between professors and managers is between 0.2 and 0.4 standard deviations, which is sizeable. The residual variance is, however, rather high among managers.

I also estimated a model with a more detailed set of professional occupations. Besides professors, this set includes teachers, engineers, medical doctors, computer programmers, accountants, architects/town and traffic planners, lawyers and judges, and artists. Also the class of managers is disaggregated, in legislators/managers of special interest groups, managers of large organizations/CEOs, and managers in small and medium enterprises. This is a particularly relevant exercise with regard to the variance, because a more fair comparison of dispersion of attitudes would compare professors with other specific occupational groups, rather than a general (possibly highly dispersed) occupational category.

As Figure 2 shows, professors are more left‐leaning than all other professional groups except one (artists), on three of the four indicators (except further European integration, where, besides artists, also the architects and planners have similar attitudes as professors). The differences are quite sizable, often between 0.2 and 0.3 standard deviations. Also, teachers stand out as an occupational group with left‐wing orientations, but only on traditional left‐right issues (income redistribution, including a very low variance around the group mean). Professional occupational groups that appear to be comparatively the most conservative/right‐wing are engineers and accountants, where the engineers also have a relatively low residual variance (i.e., little within‐occupation dispersion). Also CEOs, SME managers, and people working in the armed forces are typically right‐wing and conservative, although there is a lot of variance around their group means. These patterns correspond well to the classification of occupational groups by economic and cultural capital.

When we examine the residual variance, there is little indication that professors have an exceptionally low level of dispersion. There is little evidence for the claim that universities are left‐wing bastions where there is no room for diversity of political orientation.

Figure 3 shows the results by the field of study in which people obtained their highest degree. This allows us to compare professors with other professionals who graduated from similar fields of study. In the humanities and arts, professors position themselves more to the political left than other professionals with a humanities degree, are more supportive of immigration, and are more in favor of further European integration. Among graduates with a qualification in the social sciences—the other field that is often considered a left‐wing bastion—there are hardly any differences among professors and other professionals; only with regard to immigration social science professors are slightly more liberal than other social science graduates.

There is one political orientation indicator on which professors across all fields are more liberal than other professionals and managers within the same field of study: tolerance to immigration. This holds especially for the fields that are *not* typically considered left‐wing bastions: the medical field, economics/business, the STEM fields, and law. For these fields the residual variance is also smaller among professors than among other professionals, in line with the left‐wing bastion hypothesis. For the other outcome variables the residual variance among professors is sometimes lower, sometimes higher, without much systematic pattern. For instance, the residual variance on economic redistribution attitudes and European integration is relatively low among medical and law professors compared to other professionals with the same field of study. The exceptional position of immigration attitudes may speculatively be explained by the highly international character of academia.

## 4 DISCUSSION

The evidence for the left‐wing bastion hypothesis is mixed. Overall, when we examine the average positions of different occupational groups, we see a pattern that is in line with sociological theories positing a central role to occupations for the formation of life styles and political orientations. In particular, we find evidence for a relationship between the dominance of cultural and economic capital for the political orientation of occupational groups. Professors and artists stand out as having a more left‐wing/liberal orientation than most other professions (as can be predicted from their dominance of cultural capital), and especially CEOs and small business managers stand out as more conservative and right‐wing (fitting the dominance of economic capital in these occupational groups). Also in line with this idea is the average position taken by engineers and medical doctors, groups for which none of the two types of capital can be considered dominant according to Bourdieu (1984).

However, with regard to the residual variance, as a measure of homogeneity within occupational groups, the pattern is less clear. Professors do not stand out as having a low dispersion of orientations. If universities were exclusionary organizations where diversity of opinions is undesired and conservative scholars are excluded, one would expect this would have resulted in a high level of homogeneity of opinions. The fact that that seems not to be very clearly the case is reassuring for the contemporary debates on ideological diversity in higher education.

Also, when we split out the results by fields of study, professors with a humanities degree are more left‐wing and liberal on most indicators, but this is not the case for professors with a social science degree. Importantly, professors are more tolerant to immigration than other professional graduates of three fields that are usually not seen as left‐wing bastions: the (para‐) medical field, economics/business, the STEM fields, and law. This finding fits less well with the “conflict of the faculties” noticed by Bourdieu (1988); the professoriate is comparatively more liberal in the powerful fields (compared to non‐professors). So, overall graduates from the humanities and social sciences may be more left‐wing and liberal (professors or not), but this is not an organizational feature of the universities, as Bourdieu seems to have suggested.

A common explanation for the more liberal orientation of professionals (including professors) relative to managers concerns their attachment to education. While the evidence is mixed on the question whether education has a causal effect on political orientations (Cavaillé & Marshall, 2019; Hillygus, 2005; Lancee & Sarrasin, 2015) people with higher levels of education, and educated in the social sciences and humanities, identify typically more strongly as left‐wing in the political sphere. The more left‐leaning orientation of more educated individuals is consistent with several well‐known explanations for the formation of political values. These include the theory that intelligence is an important driver of occupational group differences in political orientation (especially on cultural issues, Carl, 2015), the theory that education socializes values especially in the social and humanistic sciences (Stubager, 2008), and the theory that consensus in orientations results from scientific wisdom rather than bias (Fuller & Geide‐Stevenson, 2014).

To further illustrate the relevance of education, it is worth emphasizing that we used model predictions of occupational group differences for respondents with a college degree, and almost all predicted outcomes are above zero (indicating above the overall average on the *z*‐standardized variables), except for the support for economic redistribution. Also managers and workers in other social classes with a college degree have above‐average scores on a liberal and left‐wing political orientation.

While we cannot test the theory that conservatives are reluctant to take up an academic career because of their atypical political orientations, the results point to the possibility that universities form an unwelcoming environment to conservatives (Gross, 2013; Rothman, Lichter, & Nevitte, 2005). Even if there is the same level of variability within the universities as elsewhere, the mean difference implies that societies’ intellectuals responsible for teaching the next generation have a more left‐leaning orientation than the rest of society. Whether this is worrying from an educational perspective is yet another question—there is no evidence that professors bring their political orientation into the classroom.

## Data Availability

I make use of the European Social Surveys (ESS). The ESS data have been collected biannually since 2002 (as a repeated cross‐sectional study), and is part of the core European Research Infrastructure funded by the European Commission. We use rounds 1–8 for this analysis (the Cumulative File rounds 1–7 edition 1 to which the round 8 Integrated File edition 2.0 has been added), and selected respondents aged 25–65 with a valid occupation code (ESS, 2016, 2018).

## NOTES

1 For instance, in the Netherlands the Royal Dutch Academy of Sciences published a report commissioned by parliament that investigates academic freedom after concerns were raised about the lack of diversity in universities (KNAW 2018).

2 Source: https://www.vsnu.nl/f_c_verhouding_vast_tijdelijk.html, last accessed August 30, 2018.

3 Source: https://www.vereniginghogescholen.nl/kennisbank/feiten-en-cijfers/artikelen/zelf-selecties-maken, last accessed August 30, 2018.

4 Source: Statistics Netherlands, http://www.cbs.nl, last accessed August 30, 2018.

5 There are 773 college graduates with a degree in personal care services in the analytical sample, of which two are in the professor category. The three most common occupations for this category were hairdressers/beauticians (*n* = 68), cooks (*n* = 92) and market salespersons/demonstrators (*n* = 112).

6 One may question whether a control for age and gender are appropriate, as the critique to universities as a left‐wing bastion is independent of the demographics of the teaching corpus. I ran models without a control for age and gender and the results were very similar. It is preferable to control for demographics as we do not want the comparison to other occupational groups to be driven by structural change in the occupational distributions.

7 Because of the weighting procedure, the weighted *N* can deviate from the weighted *N* based on sampling weights as seen in descriptive statistics.

## APPENDIX 1

**Table A1** Estimates of regression models underlying Figure 1

**Table:**

	Right‐left self‐placement		Gov’t should reduce income differences		Tolerant to immigration		Further European integration	
	Mean model	Variance function regression	Mean model	Variance function regression	Mean model	Variance function regression	Mean model	Variance function regression
Occupational group (base category: professors)								
Other professionals	−0.203***	0.074~	−0.086**	−0.059	−0.244***	0.067~	−0.191***	−0.066
	(0.027)	(0.045)	(0.029)	(0.044)	(0.023)	(0.040)	(0.034)	(0.041)
Managers	−0.427***	0.076~	−0.299***	0.069	−0.336***	0.164***	−0.198***	−0.038
	(0.028)	(0.045)	(0.030)	(0.044)	(0.024)	(0.041)	(0.034)	(0.042)
Other	−0.303***	0.056	−0.010	−0.123**	−0.461***	0.195***	−0.312***	−0.036
	(0.027)	(0.044)	(0.029)	(0.043)	(0.023)	(0.040)	(0.033)	(0.041)
Education (base category: less than lower secondary)								
Lower secondary	−0.022*	0.018	0.016~	−0.014	0.109***	−0.024~	−0.019	−0.052***
	(0.010)	(0.017)	(0.009)	(0.016)	(0.010)	(0.014)	(0.013)	(0.016)
Upper secondary	−0.005	0.035*	−0.043***	0.040**	0.286***	−0.113***	0.049***	−0.071***
	(0.009)	(0.015)	(0.008)	(0.014)	(0.009)	(0.013)	(0.012)	(0.014)
Some post‐secondary	0.049***	−0.001	−0.112***	0.097***	0.446***	−0.225***	0.116***	−0.074***
	(0.013)	(0.021)	(0.012)	(0.021)	(0.012)	(0.019)	(0.016)	(0.020)
Degree	0.047***	0.051**	−0.213***	0.192***	0.595***	−0.224***	0.215***	−0.047**
	(0.010)	(0.016)	(0.009)	(0.015)	(0.009)	(0.014)	(0.012)	(0.015)
Male (base category: female)	−0.045***	0.097***	−0.105***	0.136***	0.068***	0.075***	0.072***	0.181***
	(0.004)	(0.007)	(0.004)	(0.006)	(0.004)	(0.006)	(0.005)	(0.006)
Age	0.004**	−0.009***	−0.001	0.008***	0.008***	0.007**	−0.007***	−0.001
	(0.002)	(0.003)	(0.001)	(0.002)	(0.001)	(0.002)	(0.002)	(0.002)
Age squared	−0.000***	0.000***	0.000***	−0.000***	−0.000***	−0.000**	0.000**	0.000*
	(0.000)	(0.000)	(0.000)	(0.000)	(0.000)	(0.000)	(0.000)	(0.000)
Country fixed effects (base category: Netherlands)	Yes	Yes	Yes	Yes	Yes	Yes	Yes	Yes
ESS round (base category: first available ESS round)	Yes	Yes	Yes	Yes	Yes	Yes	Yes	Yes
Constant	0.249***	−0.231**	−0.417***	−0.156*	0.052	−1.073***	0.388***	−0.349***
	(0.045)	(0.075)	(0.045)	(0.072)	(0.040)	(0.067)	(0.055)	(0.068)
Observations	199,375	199,375	231,316	231,316	231,555	231,555	150,616	150,616

~*p* < .10, **p* < .05, ***p* < .01, ****p* < .001 (two‐tailed). Standard errors in parentheses.

**Table A2** Estimates of regression models underlying Figure 2

**Table:**

	Right‐left self‐placement		Gov’t should reduce income differences		Tolerant to immigration		Further European integration	
	Mean model	Variance function regression	Mean model	Variance function regression	Mean model	Variance function regression	Mean model	Variance function regression
Occupational group (base category: professors)								
Teachers	−0.147***	0.052	0.036	−0.157***	−0.246***	0.061	−0.219***	−0.080~
	(0.028)	(0.046)	(0.030)	(0.045)	(0.024)	(0.042)	(0.034)	(0.042)
Engineers	−0.336***	−0.075	−0.222***	−0.070	−0.308***	0.030	−0.295***	−0.197**
	(0.042)	(0.072)	(0.048)	(0.071)	(0.039)	(0.066)	(0.054)	(0.068)
Medical doctors	−0.320***	0.153*	−0.234***	−0.002	−0.264***	0.066	−0.153***	−0.064
	(0.037)	(0.059)	(0.039)	(0.058)	(0.031)	(0.054)	(0.044)	(0.054)
Computer programmers	−0.253***	0.072	−0.234***	−0.024	−0.200***	0.002	−0.162***	−0.095~
	(0.033)	(0.054)	(0.036)	(0.054)	(0.028)	(0.050)	(0.040)	(0.050)
Accountants	−0.380***	−0.049	−0.227***	0.023	−0.417***	0.099~	−0.241***	0.002
	(0.034)	(0.058)	(0.037)	(0.056)	(0.030)	(0.052)	(0.043)	(0.052)
Architects and planners	−0.202***	0.068	−0.232***	0.003	−0.172***	0.121~	−0.004	−0.083
	(0.047)	(0.076)	(0.051)	(0.076)	(0.041)	(0.070)	(0.057)	(0.070)
Lawyers and judges	−0.285***	0.167**	−0.276***	0.098	−0.252***	0.006	−0.109*	−0.197***
	(0.040)	(0.063)	(0.042)	(0.062)	(0.033)	(0.058)	(0.045)	(0.057)
Artists	0.027	0.064	0.023	−0.064	0.008	0.062	−0.041	−0.064
	(0.036)	(0.059)	(0.038)	(0.058)	(0.031)	(0.054)	(0.043)	(0.054)
Other professionals	−0.224***	0.082~	−0.128***	−0.039	−0.253***	0.067	−0.195***	−0.050
	(0.028)	(0.046)	(0.030)	(0.045)	(0.024)	(0.041)	(0.034)	(0.042)
Legislators/managers special interest group	−0.420***	0.228**	−0.223***	−0.026	−0.125**	0.168*	−0.069	−0.047
	(0.056)	(0.085)	(0.056)	(0.085)	(0.048)	(0.079)	(0.067)	(0.081)
Managers of large organizations/CEOs	−0.402***	0.039	−0.327***	0.079~	−0.306***	0.135**	−0.173***	−0.046
	(0.028)	(0.046)	(0.031)	(0.045)	(0.024)	(0.042)	(0.035)	(0.043)
Managers SMEs	−0.475***	0.127**	−0.261***	0.059	−0.405***	0.202***	−0.256***	−0.028
	(0.029)	(0.048)	(0.032)	(0.047)	(0.025)	(0.044)	(0.036)	(0.044)
Armed forces	−0.522***	0.103	−0.194***	−0.045	−0.413***	0.159*	−0.317***	0.013
	(0.043)	(0.069)	(0.045)	(0.068)	(0.039)	(0.063)	(0.054)	(0.063)
Other occupations	−0.302***	0.057	−0.005	−0.125**	−0.464***	0.197***	−0.314***	−0.036
	(0.027)	(0.044)	(0.029)	(0.043)	(0.023)	(0.040)	(0.033)	(0.041)
Education (base category: less than lower secondary)								
Lower secondary	−0.023*	0.015	0.016~	−0.014	0.108***	−0.023	−0.020	−0.051**
	(0.010)	(0.017)	(0.009)	(0.016)	(0.010)	(0.014)	(0.013)	(0.016)
Upper secondary	−0.007	0.033*	−0.044***	0.042**	0.285***	−0.112***	0.049***	−0.070***
	(0.009)	(0.015)	(0.008)	(0.014)	(0.009)	(0.013)	(0.012)	(0.014)
Some post‐secondary	0.046***	−0.003	−0.116***	0.102***	0.444***	−0.221***	0.116***	−0.072***
	(0.013)	(0.021)	(0.012)	(0.021)	(0.012)	(0.019)	(0.016)	(0.020)
Degree	0.046***	0.050**	−0.209***	0.190***	0.591***	−0.221***	0.212***	−0.044**
	(0.010)	(0.016)	(0.009)	(0.015)	(0.009)	(0.014)	(0.012)	(0.015)
Male (base category: female)	−0.040***	0.094***	−0.095***	0.129***	0.066***	0.077***	0.069***	0.181***
	(0.004)	(0.007)	(0.004)	(0.007)	(0.004)	(0.006)	(0.005)	(0.006)
Age	0.004*	−0.009***	−0.002	0.008***	0.008***	0.007**	−0.007***	−0.001
	(0.002)	(0.003)	(0.001)	(0.002)	(0.001)	(0.002)	(0.002)	(0.002)
Age squared	−0.000***	0.000***	0.000***	−0.000***	−0.000***	−0.000**	0.000**	0.000*
	(0.000)	(0.000)	(0.000)	(0.000)	(0.000)	(0.000)	(0.000)	(0.000)
Country fixed effects (base category: Netherlands)	Yes	Yes	Yes	Yes	Yes	Yes	Yes	Yes
ESS round (base category: first available ESS round)	Yes	Yes	Yes	Yes	Yes	Yes	Yes	Yes
Constant	0.255***	−0.231**	−0.414***	−0.158*	0.057	−1.073***	0.392***	−0.347***
	(0.045)	(0.075)	(0.045)	(0.072)	(0.040)	(0.067)	(0.055)	(0.068)
Observations	199,375	199,375	231,316	231,316	231,555	231,555	150,616	150,616

~*p* < .10, **p* < .05, ***p* < .01, ****p* < .001 (two‐tailed). Standard errors in parentheses.

**Table A3** Estimates of regression models underlying Figure 3

**Table:**

	Right‐left self‐placement		Gov’t should reduce income differences		Tolerant to immigration		Further European integration	
	Mean model	Variance function regression	Mean model	Variance function regression	Mean model	Variance function regression	Mean model	Variance function regression
Occupational group (base category: professors)								
Other professionals	−0.653***	0.108	−0.834***	1.347***	−0.522**	0.279	−0.989***	0.200
	(0.191)	(0.328)	(0.130)	(0.373)	(0.192)	(0.321)	(0.208)	(0.263)
Managers	−0.938***	0.284	−1.013***	1.560***	−0.706***	0.321	−1.079***	0.204
	(0.190)	(0.325)	(0.128)	(0.371)	(0.190)	(0.319)	(0.206)	(0.260)
Other	−0.660***	0.197	−0.741***	1.293***	−0.740***	0.333	−1.023***	0.210
	(0.187)	(0.322)	(0.124)	(0.368)	(0.188)	(0.316)	(0.204)	(0.257)
Field of study (base category: General/No specific field)								
Humanities/Arts	−0.187	0.296	−0.589***	1.662***	−0.225	0.358	−0.625**	0.161
	(0.206)	(0.348)	(0.155)	(0.388)	(0.203)	(0.337)	(0.219)	(0.276)
STEM	−0.423*	−0.064	−0.868***	1.616***	−0.343~	−0.264	−0.989***	0.097
	(0.202)	(0.353)	(0.159)	(0.391)	(0.198)	(0.341)	(0.220)	(0.279)
Social Sciences	−0.554**	0.272	−0.915***	1.443***	−0.342~	−0.172	−1.010***	−0.063
	(0.203)	(0.346)	(0.151)	(0.389)	(0.198)	(0.339)	(0.216)	(0.278)
(Para‐) Medical	−0.727**	−0.181	−1.013***	0.822~	−0.089	−0.886~	−0.469*	−1.315***
	(0.244)	(0.460)	(0.205)	(0.497)	(0.217)	(0.453)	(0.224)	(0.369)
Economics/Business	−0.802**	0.889*	−1.327***	1.995***	−0.209	−0.222	−0.801**	−0.163
	(0.269)	(0.396)	(0.224)	(0.422)	(0.213)	(0.374)	(0.245)	(0.333)
Legal	−0.704~	0.337	−0.848***	0.379	0.008	−0.872	−0.076	−1.148*
	(0.359)	(0.555)	(0.219)	(0.579)	(0.237)	(0.536)	(0.259)	(0.456)
Occupational group × Field of study								
Other professionals × Humanities/Arts	0.444*	−0.210	0.685***	−1.692***	0.364~	−0.355	0.738***	−0.256
	(0.211)	(0.357)	(0.162)	(0.395)	(0.208)	(0.344)	(0.224)	(0.283)
Other professionals × STEM	0.412*	0.085	0.732***	−1.561***	0.241	0.165	1.048***	−0.163
	(0.206)	(0.360)	(0.165)	(0.397)	(0.202)	(0.348)	(0.225)	(0.284)
Other professionals × Social Sciences	0.647**	−0.150	0.997***	−1.446***	0.362~	−0.008	1.045***	−0.044
	(0.207)	(0.353)	(0.157)	(0.395)	(0.202)	(0.345)	(0.221)	(0.283)
Other professionals × (Para‐) Medical	0.698**	0.397	0.866***	−0.690	0.058	0.760~	0.597**	1.197**
	(0.248)	(0.466)	(0.210)	(0.503)	(0.222)	(0.459)	(0.229)	(0.374)
Other professionals × Economics/Business	0.653*	−0.790~	1.102***	−1.872***	0.114	0.138	0.843***	0.177
	(0.273)	(0.404)	(0.229)	(0.429)	(0.217)	(0.381)	(0.250)	(0.338)
Other professionals × Legal	0.658~	−0.190	0.603**	−0.189	−0.021	0.821	0.220	1.113*
	(0.364)	(0.563)	(0.227)	(0.585)	(0.243)	(0.543)	(0.266)	(0.462)
Managers × Humanities/Arts	0.488*	−0.251	0.600***	−1.684***	0.477*	−0.397	0.771***	−0.236
	(0.213)	(0.360)	(0.166)	(0.397)	(0.210)	(0.347)	(0.226)	(0.285)
Managers × STEM	0.517*	−0.005	0.782***	−1.661***	0.377~	0.200	1.122***	−0.150
	(0.206)	(0.358)	(0.164)	(0.395)	(0.201)	(0.346)	(0.223)	(0.283)
Managers × Social Sciences	0.751***	−0.295	0.971***	−1.569***	0.515*	0.122	1.277***	0.021
	(0.208)	(0.353)	(0.158)	(0.395)	(0.202)	(0.345)	(0.221)	(0.284)
Managers × (Para‐) Medical	1.042***	0.109	1.002***	−0.984~	0.290	0.752	0.593*	1.232**
	(0.252)	(0.472)	(0.216)	(0.508)	(0.226)	(0.464)	(0.233)	(0.379)
Managers × Economics/Business	0.795**	−0.913*	1.024***	−1.968***	0.276	0.276	0.958***	0.131
	(0.272)	(0.401)	(0.228)	(0.427)	(0.216)	(0.379)	(0.249)	(0.337)
Managers × Legal	0.545	−0.223	0.805***	−0.367	0.062	0.774	0.266	0.999*
	(0.368)	(0.566)	(0.232)	(0.589)	(0.246)	(0.546)	(0.269)	(0.465)
Other × Humanities/Arts	0.219	−0.162	0.578***	−1.542***	0.439*	−0.434	0.700**	−0.184
	(0.207)	(0.350)	(0.157)	(0.389)	(0.204)	(0.338)	(0.220)	(0.278)
Other × STEM	0.390~	0.024	0.863***	−1.576***	0.257	0.267	0.918***	−0.118
	(0.203)	(0.353)	(0.160)	(0.392)	(0.198)	(0.342)	(0.221)	(0.279)
Other × Social Sciences	0.540**	−0.298	0.927***	−1.410***	0.331~	0.153	0.978***	0.005
	(0.203)	(0.347)	(0.152)	(0.390)	(0.198)	(0.339)	(0.217)	(0.278)
Other × (Para‐) Medical	0.708**	0.195	1.004***	−0.797	0.106	0.851~	0.440~	1.179**
	(0.244)	(0.461)	(0.205)	(0.498)	(0.218)	(0.454)	(0.224)	(0.370)
Other × Economics/Business	0.689*	−0.903*	1.230***	−1.882***	0.202	0.120	0.801**	0.077
	(0.270)	(0.397)	(0.224)	(0.423)	(0.213)	(0.375)	(0.246)	(0.333)
Other × Legal	0.552	−0.339	0.677**	−0.276	−0.087	0.773	0.039	1.067*
	(0.360)	(0.557)	(0.221)	(0.580)	(0.239)	(0.538)	(0.261)	(0.458)
Educational attainment (base category: less than lower secondary)								
Lower secondary	−0.049*	0.001	0.015	0.039	0.067***	0.017	0.083***	0.001
	(0.022)	(0.034)	(0.017)	(0.031)	(0.020)	(0.030)	(0.022)	(0.027)
Upper secondary	−0.033	0.083*	−0.028	0.054~	0.265***	−0.068*	0.160***	0.023
	(0.022)	(0.034)	(0.018)	(0.031)	(0.019)	(0.030)	(0.022)	(0.027)
Some post‐secondary	0.049~	0.096*	−0.113***	0.150***	0.407***	−0.210***	0.218***	0.045
	(0.027)	(0.045)	(0.025)	(0.041)	(0.025)	(0.040)	(0.029)	(0.035)
College degree	0.027	0.077*	−0.186***	0.186***	0.541***	−0.146***	0.288***	0.024
	(0.023)	(0.036)	(0.019)	(0.033)	(0.021)	(0.032)	(0.023)	(0.029)
Male (base category: female)	−0.048***	0.146***	−0.096***	0.128***	0.101***	0.059***	0.102***	0.160***
	(0.008)	(0.013)	(0.007)	(0.012)	(0.007)	(0.012)	(0.008)	(0.010)
Age	0.016***	−0.001	0.008**	0.003	0.016***	0.004	−0.006*	−0.001
	(0.003)	(0.004)	(0.002)	(0.004)	(0.002)	(0.004)	(0.003)	(0.003)
Age squared	−0.000***	0.000*	−0.000	−0.000	−0.000***	−0.000	0.000~	0.000
	(0.000)	(0.000)	(0.000)	(0.000)	(0.000)	(0.000)	(0.000)	(0.000)
Country fixed effects (base category: Netherlands)	Yes	Yes	Yes	Yes	Yes	Yes	Yes	Yes
ESS round (base category: round 2)	Yes	Yes	Yes	Yes	Yes	Yes	Yes	Yes
Constant	0.473*	−0.676*	0.177	−1.476***	0.154	−0.998**	1.019***	−0.650*
	(0.197)	(0.338)	(0.138)	(0.379)	(0.197)	(0.329)	(0.213)	(0.269)
Observations	69,597	69,597	80,553	80,553	80,623	80,623	71,387	71,387

~*p* < .10, **p* < .05, ***p* < .01, ****p* < .001 (two‐tailed). Standard errors in parentheses.
